# Quantification and Phylogenetic Analysis of Ammonia Oxidizers on Biofilm Carriers in a Full-Scale Wastewater Treatment Plant

**DOI:** 10.1264/jsme2.ME19140

**Published:** 2020-04-03

**Authors:** Yuki Tsuchiya, Tatsunori Nakagawa, Reiji Takahashi

**Affiliations:** 1 College of Bioresource Sciences, Nihon University, 1866 Kameino, Fujisawa, Kanagawa, 252–0880, Japan

**Keywords:** ammonia-oxidizing bacteria, comammox, biofilm, WWTP, real-time PCR

## Abstract

Biofilm carriers have been used to remove ammonia in several wastewater treatment plants (WWTPs) in Japan. However, the abundance and species of ammonia oxidizers in the biofilms formed on the surface of carriers in full-scale operational WWTP tanks remain unclear. In the present study, we conducted quantitative PCR and PCR cloning of the *amoA* genes of ammonia-oxidizing bacteria and archaea (AOB and AOA) and a complete ammonia oxidizer (comammox) in the biofilm formed on the carriers in a full-scale WWTP. The quantification of *amoA* genes showed that the abundance of AOB and comammox was markedly greater in the biofilm than in the activated sludge suspended in a tank solution of the WWTP, while AOA was not detected in the biofilm or the activated sludge. A phylogenetic analysis of *amoA* genes revealed that as-yet-uncultivated comammox *Nitrospira* and uncultured AOB *Nitrosomonas* were predominant in the biofilm. The present results suggest that the biofilm formed on the surface of carriers enable comammox *Nitrospira* and AOB *Nitrosomonas* to co-exist and remain in the full-scale WWTP tank surveyed in this study.

Nitrification, the biological oxidation process by which ammonia is converted to nitrite then nitrate, is an important nitrogen removal step in wastewater treatment plants (WWTPs). Nitrification is considered to be driven by two phylogenetically different groups of chemolithotrophic microorganisms: ammonia-oxidizing bacteria and archaea (AOB and AOA, respectively) and nitrite-oxidizing bacteria (NOB). A complete ammonia oxidizer (comammox) that converts ammonia to nitrate in one cell was recently discovered in the NOB genus *Nitrospira* in enrichment cultures of biofilms that formed on the wall of a hot-water pipe (Daims *et al.*, 2015) and on the trickling filter of an aquaculture system (van Kessel *et al.*, 2015). AOB and AOA are key microorganisms influencing the rate of nitrogen removal in WWTPs due to their slow growth rates (Limpiyakorn *et al.*, 2005; 2013). Moreover, comammox *Nitrospira* was detected in the activated sludge of several WWTPs using high-throughput sequencing (Pjevac *et al.*, 2017; Wang *et al.*, 2018; Roots *et al.*, 2019) and is recognized as an important member for nitrification in WWTPs (Chao *et al.*, 2016; Annavajhala *et al.*, 2018). The community composition of ammonia oxidizers (AOB, AOA, and comammox *Nitrospira*) and their influence on nitrification in WWTPs are being highlighted worldwide.

Advanced systems using activated sludge, membrane bioreactors, or biofilm carriers are adopted by many full-scale WWTPs for nitrogen removal from wastewater. In these systems, the biofilm carrier system has potential to achieve efficient (low cost and quick) nitrification in WWTPs (Wang *et al.*, 2005; Chung *et al.*, 2007) because biofilm-forming AOB exhibit higher affinity for ammonium and faster recovery after ammonium starvation than planktonic cells (Batchelor *et al.*, 1997; Bollmann *et al.*, 2005). Moreover, other studies at a laboratory-scale reactor and pilot plant using biofilm carriers demonstrated that biofilm carriers successfully retained AOB and easily maintained nitrification activity (Welander *et al.*, 1997; Mishima *et al.*, 1998; Young *et al.*, 2016). However, limited information is currently available on ammonia oxidizers (AOB, AOA, and comammox *Nitrospira*) on biofilm carriers in operational full-scale WWTPs. Thus, detailed investigations on the abundance and phylogeny of ammonia oxidizers on biofilm carriers will lead to a better understanding of the ecology of ammonia oxidizers in WWTPs. Furthermore, useful information will be obtained to resolve nitrification failure (Kim *et al.*, 2006; Tang and Chen, 2015), which sometimes occurs in WWTPs.

In the present study, we conducted quantitative PCR (qPCR) and sequencing of the *amoA* genes of each ammonia oxidizer (AOB, AOA, and comammox) on biofilm carriers collected from an advanced full-scale operational WWTP in the Kanto area of Japan, and discussed the contribution of these ammonia oxidizers to nitrification in the WWTP.

## Materials and Methods

### Sample collection

**Fig. 1.**
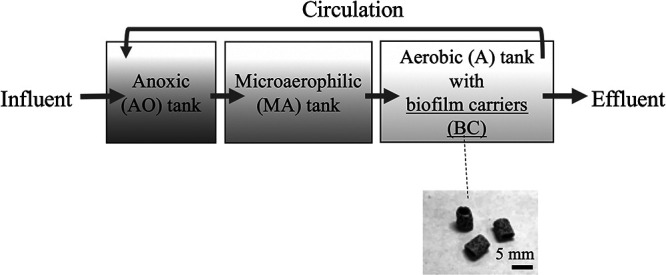
Schematic illustration of the full-scale WWTP in the present study. Solution containing activated sludge was collected from an anoxic (AO) tank and microaerophilic (MA) tank. A solution containing activated sludge and biofilm carriers (BC) was collected from an aerobic (A) tank.

Biofilm carrier samples were collected from an advanced WWTP in the Kanto area of Japan on 23 February, 2018. This particular WWTP serves a population of approximately 2,000,000 and treats 800,000 m^3^ of full-scale wastewater daily. The plant consists of an anoxic tank (AO-tank), microaerophilic tank (MA-tank), and aerobic tank (A-tank) in that order for the treatment of sewage (Fig. 1). Ring-shaped elements (approximately 4‍ ‍mm in diameter and 5‍ ‍mm in length) made of polypropylene (Bio-Tube, JFE Engineering) are suspended in the A-tank to act as a biofilm carrier (BC). Some of the treated solution from the A-tank is circulated into the AO-tank.

Approximately 100‍ ‍mL of solution containing BC was collected from the A-tank. Suspended activated sludge solution samples (100‍ ‍mL each) were also collected from the AO-, MA-, and A-tanks. These samples were brought back to the laboratory inside a cooler set at *ca.* 4°C. Immediately after arriving at the laboratory, activated sludge solution samples were centrifuged (8,000×*g*, 4°C, 10‍ ‍min) and the supernatant and sediment were separated. The supernatant was filtered (0.22 μm, Millex-GV: Merck Millipore) and used to measure the concentrations of NH_4_^+^, NO_2_^–^, and NO_3_^–^ ions and disolved saccharides and proteins. The sediment containing activated sludge was stored at –‍80°C until used for DNA extraction; these activated sludge samples contained planktonic cells. The BC sample was gently washed 3 times with the supernatant of the A-tank solution to remove extra activated sludge, and then stored at –80°C until used in the DNA extraction process.

### Sample characteristics

The concentrations of NO_2_^–^ and NO_3_^–^ ions in the supernatant of the tank solution collected from the AO-, MA-, and A-tanks were measured by ion chromatography (Dionex ICS-1000). The identification of NO_2_^–^ and NO_3_^–^ peaks was conducted using external standard methods. The concentration of NH_4_^+^ was measured by the indophenol blue method (Sagi, 1966). The concentrations of dissolved saccharides and proteins were measured by the phenol-sulfuric acid method (Kochert, 1978) and Bradford method (Bradford, 1976), respectively. Light absorbance was measured using a spectrophotometer (U-5100; HITACHI). Each measurement was conducted in triplicate.

### DNA extraction

The BC samples collected from the A-tank and activated sludge samples collected from each tank (AO, MA, and A) were precisely weighed (*ca.* 0.2‍ ‍g and 0.1‍ ‍g for BC and activated sludge, respectively), and DNA in the samples was then extracted with the ISOIL for Beads Beating Kit (Nippon Gene) according to the manufacturer’s instructions. Four carriers (*ca.* 0.2‍ ‍g containing *ca.* 0.04‍ ‍g of the biofilm) were broken with sterilized tweezers and directly used for DNA extraction. Extracted DNA was suspended in 50‍ ‍μL of TE buffer (attached in the ISOIL for Beads Beating Kit) and stored at –20°C until qPCR. DNA extraction was conducted in triplicate.

### qPCR of AOB *amoA*, comammox *amoA*, and 16S rRNA genes

AOB *amoA*, AOA *amoA*, comammox *amoA*, and 16S rRNA gene copies were quantified by real-time PCR with the primers amoA 1F and 2R (Rotthauwe *et al.*, 1997), GenAOA F and -R (Meinhardt *et al.*, 2015), Ntsp-amoA 162F and 359R (Fowler *et al.*, 2018), and EUB 926F and 1092R (De Gregoris *et al.*, 2011), respectively (Table 1). Triplicate DNA samples were used for real-time PCR performed with the CFX 96 Real-Time System (Bio-Rad). The PCR mixture was prepared to a total volume of 25‍ ‍μL and contained 2‍ ‍μL of 10- to 50-fold diluted DNA sample, 5 pmol of each primer, and 12.5‍ ‍μL of the SYBR Premix Ex Taq kit (Takara Bio). The PCR program for the AOB *amoA* gene consisted of 94°C for 5‍ ‍min followed by 35 cycles at 94°C for 30‍ ‍s, at 55°C for 30‍ ‍s, and at 72°C for 30 s. The program for the comammox *amoA* gene was as follows: at 95°C for 3‍ ‍min followed by 35 cycles at 95°C for 10‍ ‍s, at 57°C for 30‍ ‍s, and at 72°C for 30 s. Regarding the 16S rRNA gene, the program was as follows: at 95°C for 5‍ ‍min followed by 35 cycles at 95°C for 15‍ ‍s, at 61.5°C for 15‍ ‍s, and at 72°C for 20 s. The specificity of qPCR products was checked by a melting curves analysis and agarose gel electrophoresis. Amplification efficiency ranged between 81 and 95% with R^2^ values higher than 0.990 for all calibration curves.

The copy number of the gene was calculated with a standard curve generated from a series of 10-fold dilutions (10^1^–10^8^) of the plasmid, with each containing cloned genes (the AOB *amoA* gene fragment of *Nitrosomonas stercoris* KYUHI-S^T^ [Nakagawa and Takahashi, 2015], and comammox *amoA* of the environmental clone retrieved from the present study [clone name, BC-COMA15; Accession number in DDBJ, LC503646]). Plasmid DNAs were prepared using the TA cloning method as shown previously (Nakagawa *et al.*, 2019). Briefly, plasmids containing the target genes were constructed by cloning PCR products into the TOPO vector using the TOPO TA Cloning Kit (Invitrogen). Plasmids were extracted using the Quantum Prep Plasmid Miniprep Kit (BioRad), and DNA concentrations were measured with the Qubit dsDNA HS Assay Kit and Qubit Fluorometer (Life Technologies).

The cell numbers of AOB, comammox, and total bacteria were calculated from the gene copy numbers obtained from qPCR (AOA was not detected; see Results and Discussion). The gene copy numbers of AOB *amoA* and comammox *amoA* per genome of the bacterial cell were assumed to be 2.5 and 1.0, respectively, based on the average copy numbers of the betaproteobacterial *amoA* gene (Norton *et al.*, 2002; Wang *et al.*, 2015; Wang *et al.*, 2018) and genomic information on the comammox *amoA* gene (Daims *et al.*, 2015; Wang *et al.*, 2018). The cell number of comammox may be overestimated because comammox species that contain 2 copies of the *amoA* gene in one cell were also found (*e.g.* *Candidatus* Nitrospira nitrosa) (Camejo *et al.*, 2017). The 16S rRNA gene copy numbers per genome of the bacterial cell was assumed to be 3.6 based on the average 16S rRNA gene copies found in cultured bacteria (Klappenbach *et al.*, 2001; Limpiyakorn *et al.*, 2005).

### Cloning, sequencing, and phylogenetic analysis of AOB and comammox *amoA* genes

The *amoA* genes of AOB and comammox in the biofilm formed on BC samples were amplified by PCR using primer sets (as shown in Table 1) and Go Taq G2 Hot Start Master Mix (Promega) following the manufacturer’s instructions. PCR was performed at 95°C for 2‍ ‍min (for a hot start), then at 94°C for 2‍ ‍min followed by 33 cycles at 94°C for 30‍ ‍s, at 55°C for 30‍ ‍s, and at 72°C for 30‍ ‍s, and then at 72°C for 10‍ ‍min for the final extension. After agarose electrophoresis, amplified DNA fragments were cloned into the vector pCR2.1-TOPO with a TOPO TA PCR Cloning Kit (Life Technologies) following the manufacturer’s instructions. Positive colonies were directly amplified with the vector-specific primers M13F and M13R (Life Technologies). After checking the length of DNA using agarose gel electrophoresis, amplicons were purified with a QIA quick PCR purification kit (Qiagen) prior to sequencing. Both strands of PCR products amplified using the M13F and M13R primers were sequenced with a BigDye Terminator version 3.1 (Life Technologies) on a 3130xl Genetic Analyzer (Life Technologies), and connected into one sequence using MEGA7 (Kumar *et al.*, 2016). The sequences obtained were compared with reference sequences in the NCBI database by the BLAST program. Sequences with 97% similarity were grouped into an operational taxonomic unit (OTU). Each representative OTU (*ca.* 400 bp) was aligned with CLUSTALW and used to construct a neighbor-joining (NJ) tree with MEGA7. Bootstrap values were assessed from 1,000 replications. To compare the richness of the AOB and comammox community, a rarefaction analysis was performed with Analytic Rarefaction v1.3 software (https://strata.uga.edu/software/index.html), and Good’s coverage value (Good, 1953) was calculated.

The *amoA* sequences of AOB and comammox in the present study were deposited in the DDBJ under accession numbers LC503692 to LC503740 (AOB) and LC503632 to LC503691 (comammox) (Table S1 and S2).

### Statistical analysis

Differences in the concentrations of N-related ions and the abundance of the *amoA* and 16S rRNA genes among samples were verified using a one-way analysis of variance (ANOVA); multiple comparisons were performed using the Tukey-Kramer test in JMP v1.4 software (SAS Institute).

## Results and Discussion

### Characteristics of tanks in WWTP

**Fig. 2.**
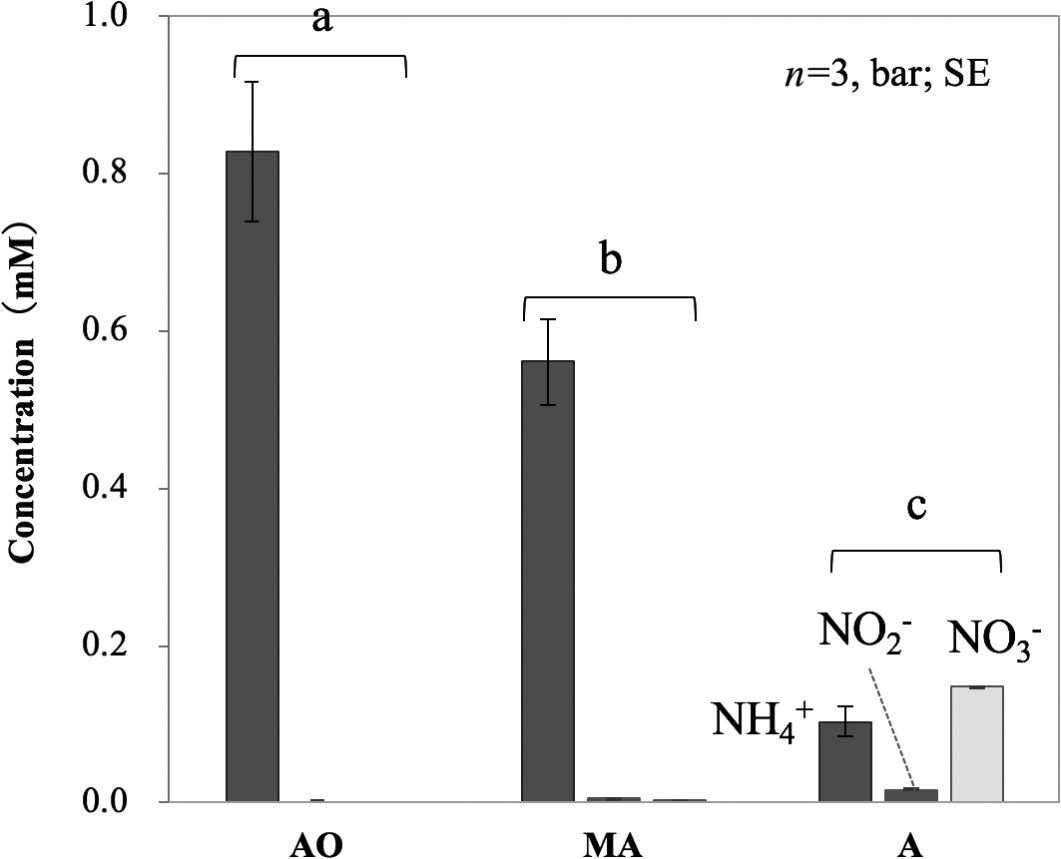
Average concentrations of NH_4_^+^, NO_2_^–^, and NO_3_^–^ in anoxic (AO), microaerophilic (MA), and aerobic (A) tanks. Different letters above the columns indicate significant differences (*P*<0.05). SE represents the standard error.

The WWTP in the present study consisted of AO-, MA-, and A-tanks with suspended BC (in that order) for the treatment of full-scale sewage (Fig. 1). The water temperature of all tanks was 19.2–19.3°C. The pH of all tanks was 6.8–7.0. The dissolved oxygen (DO) concentrations in the AO-, MA-, and A-tanks were *ca.* 0, 1.3, and 6.0‍ ‍mg L^–1^, respectively. The dissolved saccharide concentration was *ca.* 4‍ ‍mg glucose equivalent L^–1^ for all tanks. Dissolved protein was not detected in any tank. The AO-tank had the highest NH_4_^+^ concentration, followed by the MA-tank, and then the A-tank (Fig. 2). NO_2_^–^ and NO_3_^–^ concentrations significantly increased (*P*<0.01) in the MA-tank and markedly in the A-tank (Fig. 2). These results suggest that nitrification occurred in the MA- and A-tanks. The dissolved inorganic nitrogen (DIN) concentration (NH_4_^+^, NO_2_^–^, and NO_3_^–^ concentrations combined) in the AO-tank (*ca.* 830‍ ‍μM) decreased in the MA- and A-tanks (*ca.* 570 and 270‍ ‍μM, respectively), suggesting that the (aerobic) denitrification process (Liu *et al.*, 2018) and/or utilization of nitrogen also occurred in these two tanks.

### Abundance of ammonia oxidizers and total bacteria

**Fig. 3.**
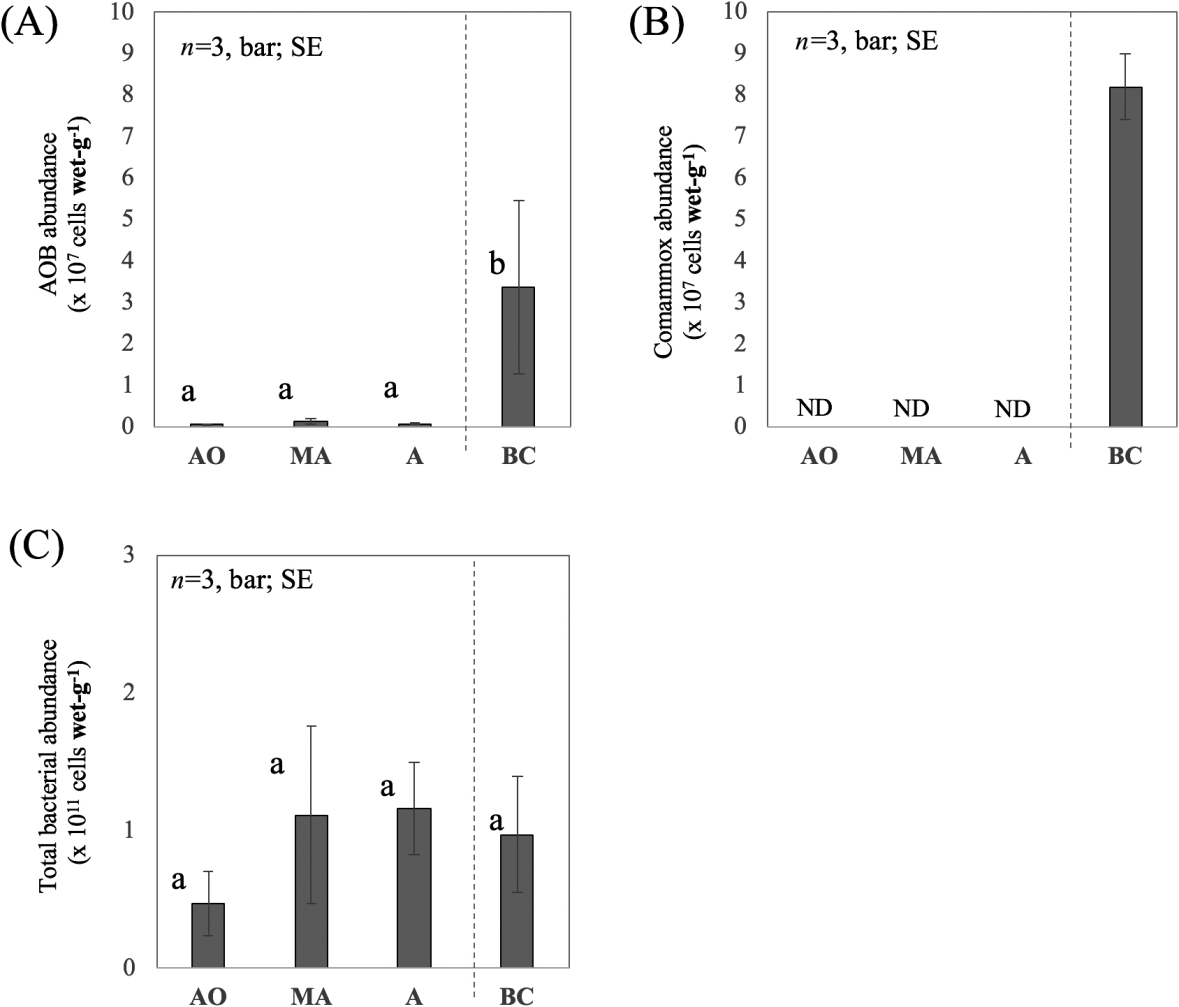
Average abundance of AOB (A), comammox (B), and total bacteria (C) in the unit wet weight of the activated sludge in AO-, MA-, and A-tanks and the biofilm on BC. Different letters above the columns indicate significant differences (*P*<0.05). The comammox in the activated sludge of the tanks was below the detection limit (shown as ND). SE represents the standard error.

We quantified the copy numbers of the *amoA* genes and 16S rRNA gene in the biofilm formed on the surface of BC of the A-tank and in the suspended activated sludge of the AO-, MA-, and A-tanks using the qPCR method with specific primer sets (Table 1) and estimated the cell numbers of ammonia oxidizers and total bacteria (Fig. 3).

The average abundance of AOB in the biofilm formed on the surface of BC was 3.4±2.2×10^7^ cells wet-g^–1^, which was significantly (*P*<0.05) greater than that in the activated sludge of all tanks (*ca.* 0.5–1.3×10^6^ cells wet-g^–1^) (Fig. 3A). The A-tank solution is continuously mixed and partially flows away. Under this condition, it is probably difficult for AOB in activated sludge to grow and remain in the tank due to slow growth. Biofilm formation on BC may be advantageous for AOB to remain in the tank, as reported in other studies (Chung *et al.*, 2007; Chao *et al.*, 2016).

Comammox with an average abundance of 8.2±0.79×10^7^ cells wet-g^–1^ was detected in the biofilm on BC (Fig. 3B). This number was higher than that of AOB in the biofilm. Based on genomic and physiological analyses, comammox appeared to adapt to low concentrations of NH_4_^+^ (*ca.* 1‍ ‍mg or less NH_4_^+^-N L^–1^) and DO (*ca.* 1‍ ‍mg L^–1^) (Daims *et al.*, 2015; Kits *et al.*, 2017; Palomo *et al.*, 2018). Therefore, comammox generally finds it challenging to compete with AOB when WWTP tanks are under eutrophic and/or aerobic conditions (Gonzalez-Martinez *et al.*, 2016; Annavajhala *et al.*, 2018; Roots *et al.*, 2019). In the A-tank of the present study, the DO concentration (*ca.* 6‍ ‍mg L^–1^) was too high for comammox, whereas the NH_4_^+^ concentration was sufficiently low (*ca.* 1‍ ‍mg NH_4_^+^-N L^–1^). In the biofilm on carriers, an environment that is suitable for comammox (low concentrations of O_2_ and NH_4_^+^) may be formed, as suggested in other studies (Lawson and Lücker, 2018; Koch *et al.*, 2019). Gradients of DO and NH_4_^+^ concentrations have been identified inside biofilms (Flemming *et al.*, 2016; Satoh *et al.*, 2006; Wang *et al.*, 2016). Biofilms may generate a niche for comammox, leading to the co-existence of comammox with AOB under the tank condition. Further analyses of 1) the spatial distribution of comammox and AOB inside the biofilm on BC and 2) the profiles of DO and ion concentrations inside the biofilm are needed in order to obtain a more detailed understanding of the relationship between comammox and AOB in biofilms.

Comammox numbers in the activated sludge of all tanks were below the detection limit (Fig. 3B). Tank solution conditions were probably inadequate for comammox, as described above. In the A-tank, an excessive DO concentration (*ca.* 6‍ ‍mg L^–1^) was observed, whereas the NH_4_^+^ concentration was sufficiently low. In the AO and MA-tanks, an excessive NH_4_^+^-N (more than 1‍ ‍mg L^–1^) concentration was detected, whereas the DO concentration was sufficiently low. These conditions may negatively affect the growth of comammox outside the biofilm, resulting in the absence of comammox (or only a small number) in the activated sludge of the tanks.

The primer set of comammox *amoA* for qPCR used in the present study was designed to detect total comammox (clades A and B) (Fowler *et al.*, 2018). A recent study reported that the comammox number might be overestimated using this primer set; extra bands in agarose gel electrophoresis and broad peaks in a melting curve analysis after qPCR were observed in some activated sludge samples (Beach and Noguera, 2019). In the present study, although weak extra bands were observed in agarose electrophoresis from BC samples, broad peaks were not detected in the melting curve analysis (Fig. S1). Thus, the primer-related overestimation of the comammox number was not significant, at least for BC samples in the present study.

The *amoA* gene of AOA was not amplified by PCR from any samples in the present study (data not shown). This result suggests no or few AOA in the biofilm and the activated sludge in the WWTP. AOA adapts to a much lower NH_4_^+^ concentration than AOB and comammox (Martens-Habbena *et al.*, 2009; Zhang *et al.*, 2015). In the present study, the NH_4_^+^ concentrations in each tank solution and in the biofilm on BC may be inadequate for AOA. Several studies also reported that AOA in WWTPs were difficult to detect using the qPCR method with the present primer sets due to their small numbers (Park *et al.*, 2006; Gao *et al.*, 2014).

The total bacterial numbers in the biofilm on BC and in the activated sludge of all tanks were in the order of 10^10^–10^11^ cells wet-g^–1^, with no significant differences being observed among samples (Fig. 3C). These numbers were similar to those in the activated sludge of other WWTPs (*ca.* 10^10^–10^11^ cells g^–1^ of MLVSS [mixed liquor volatile suspended solids]) (Harms *et al.*, 2003), which suggests similar bacterial densities on carrier biofilms and activated sludge among WWTPs.

### Relative abundance of comammox and AOB in total bacterial number

**Fig. 4.**
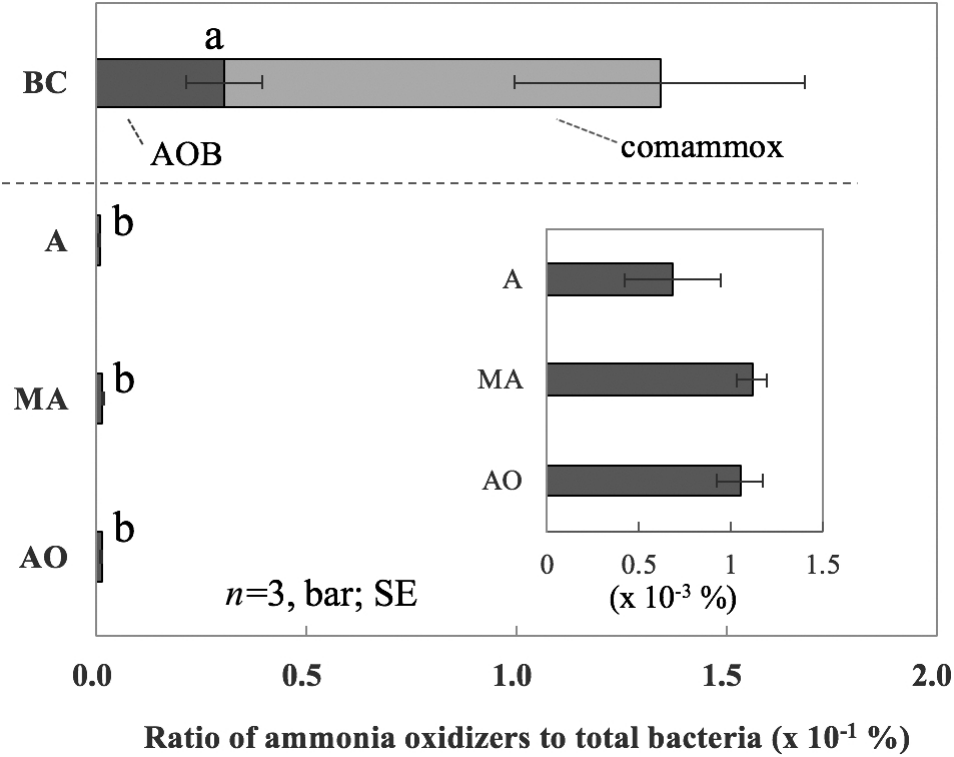
Percentages of AOB and comammox numbers in total bacterial numbers in the activated sludge of AO-, MA-, and A-tanks and the biofilm on BC. Different letters above the columns indicate significant differences (*P*<0.05). SE represents the standard error.

The percentages of the number of comammox and AOB out of the total number of bacteria were calculated (Fig. 4). The AOB number in the biofilm on BC accounted for *ca.* 3×10^–2^% of total bacteria. This percentage was significantly (*P*<0.05) higher than that in the activated sludge of the tanks (*ca.* 2–5×10^–3^%). Including comammox, the percentage of the ammonia oxidizers in the biofilm on BC increased to approximately 100-fold (*ca.* 3.5×10^–1^%) that in the activated sludge. This result suggests that comammox and AOB attach and grow on BC more easily than other bacteria. A similar percentage was reported for the activated sludge samples of other WWTPs (Wang *et al.*, 2018). Although the percentage of comammox and AOB in total bacteria was no more than 3.5×10^–1^% in the present study, ammonia oxidizers on BC may have similar nitrification potential as ammonia oxidizers in activated sludge.

### Comparison of the abundance of AOB, comammox, and total bacteria among tanks

**Fig. 5.**
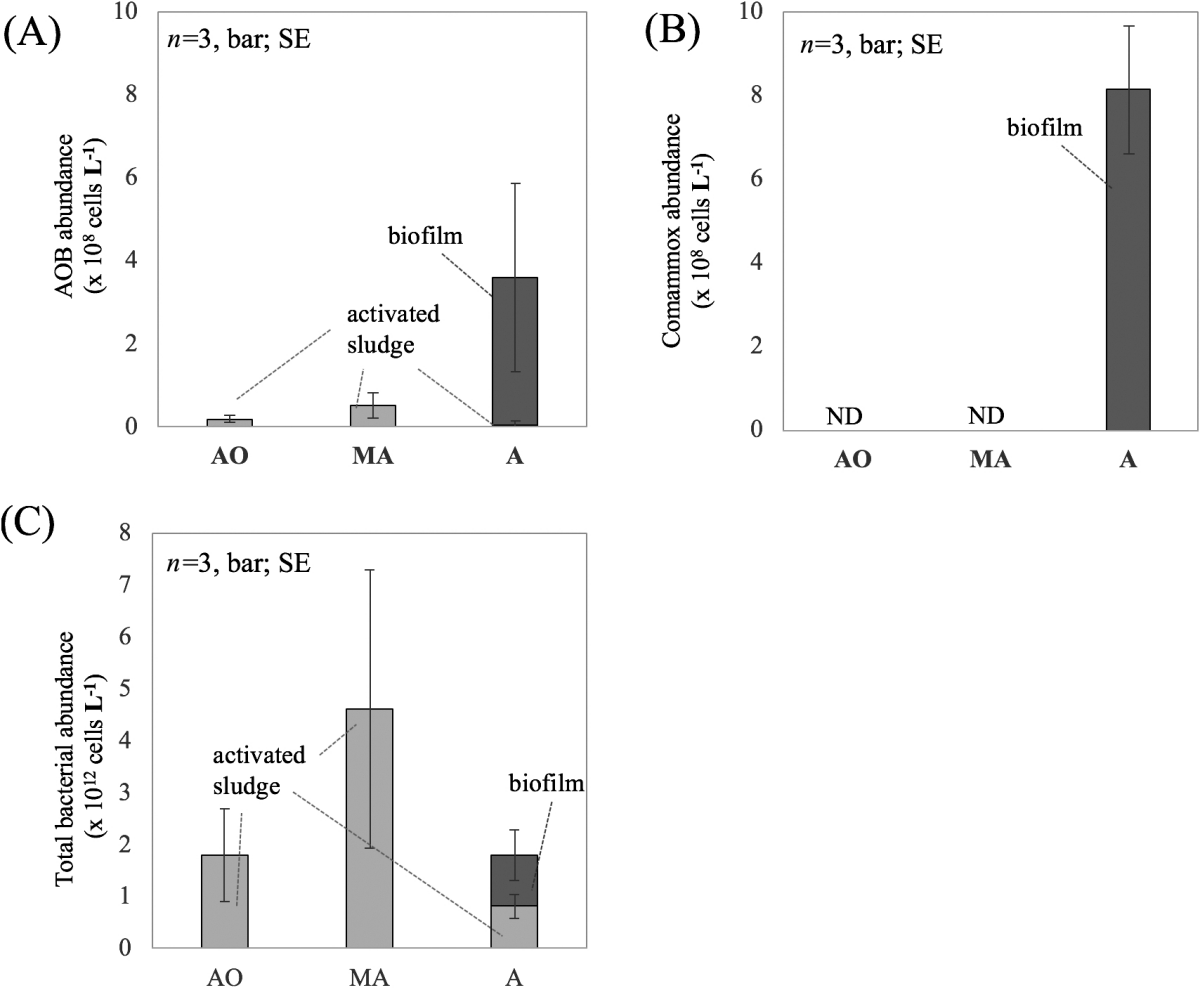
Average abundance of AOB (A), comammox (B), and total bacteria (C) in the unit volume (L) of solutions in AO-, MA-, and A-tanks, calculated based on numbers shown in Fig. 3. There were no significant differences among the samples (*P*>0.05). SE shows the standard error.

The activated sludge (and biofilm) volume in the collected tank solution differed among tanks (Table S3). To compare the abundance of bacteria among the tanks, we calculated the numbers of comammox, AOB, and total bacteria in a unit volume of the tank solution (cells L^–1^) (Fig. 5). The A-tank solution had the greatest abundance of AOB per litter among the three tank solutions (Fig. 5A). As shown in Fig. 2, decreased NH_4_^+^ and increased NO_2_^–^ and NO_3_^–^ concentrations were observed in the A-tank, in which aerobic conditions were maintained by aeration (DO was *ca.* 6‍ ‍mg L^–1^). AOB in the A-tank may make a major contribution to the nitrification of the WWTP. In particular, AOB in the biofilm on BC (accounting for more than 90% of AOB in the A-tank) may play an important role in tank nitrification.

In the MA-tank (in which the second greatest AOB abundance was observed) decreased NH_4_^+^ and increased NO_2_^–^ and NO_3_^–^ concentrations under microaerophilic conditions were observed (DO was *ca.* 3‍ ‍mg L^–1^), suggesting less activity and smaller contribution of nitrification by AOB than that in the A-tank. On the other hand, AOB in the AO-tank was probably inhibited by anaerobic conditions (DO was *ca.* 0‍ ‍mg L^–1^). The detection of AOB in the AO-tank may have been due to the recycling of the A-tank solution. Otherwise, the AOB may have originated from the influent.

The abundance of comammox per litter of A-tank solution was similar to (or higher than) AOB abundance (Fig. 5B). In addition to AOB, comammox may make a considerable contribution to nitrification in the WWTP. In contrast to AOB, comammox was only detected in the biofilm on BC. BC appears to be important for the accumulation of comammox and achieving efficient nitrification in the WWTP. As described above, the environment inside the biofilm may be adequate for comammox (low concentrations of O_2_ and NH_4_^+^). Several studies reported that the genus *Nitrospira*, containing comammox as well as canonical NOB, was located in the inner part of biofilms in order to avoid being exposed to excessive oxygen, while AOB, such as *Nitrosomonas*, was located in the outer part of biofilms due to low affinity to oxygen and NH_4_^+^ (Okabe *et al.*, 1999; Schramm *et al.*, 2000; Suarez *et al.*, 2019). This may also be the case for comammox and AOB in the biofilm on BC.

The abundance of total bacteria per liter of tank solution was in the order of 10^12^ cells L^–1^ for all tanks, with no significant differences being observed among the tanks (Fig. 5C), suggesting that the concentrations of N-related ions and DO that differed among tanks did not affect the number of total bacteria. Relatively greater abundance was observed in the MA-tank (4.6×10^12^ cells L^–1^). The dissolved saccharide concentration in the tank solution slightly decreased from the AO-tank to MA-tank (from *ca.* 4.3±0.4 to 3.8±0.2‍ ‍mg glucose equivalent L^–1^). Organic substances in the tank solution might affect the abundance of bacteria. However, further analyses of the relationship between bacterial abundance and the concentrations of organic and inorganic substances in each tank are necessary.

The abundance and activities of AOB and AOA are generally known to be influenced by many factors such as water characteristics (including ammonia concentrations and organic loading), environmental parameters (such as temperature, pH, DO concentrations, and retention times) (Almstrand *et al.*, 2011), and the type of wastewater treatment system (activated sludge, moving-bed, etc) (Koops and Pommerening-Röser, 2001; Sinthusith *et al.*, 2015; Wang *et al.*, 2018). Although the present study showed sample data collected from a WWTP at one time point, future studies need to examine the time course of the abundance of ammonia oxidizers in the WWTP and compare the contribution that each ammonia oxidizer makes to nitrification in several plants.

### Community compositions of AOB and comammox on biofilm carriers

qPCR revealed that biofilm carriers hold markedly greater numbers of AOB and comammox than activated sludge, and, thus, probably contribute more to nitrification in the WWTP. In the present study, the community composition of AOB and comammox in the biofilm was analyzed using TA cloning. Good’s coverages (Good, 1953) of AOB and comammox clone libraries were 91 and 83%, respectively.

**Fig. 6.**
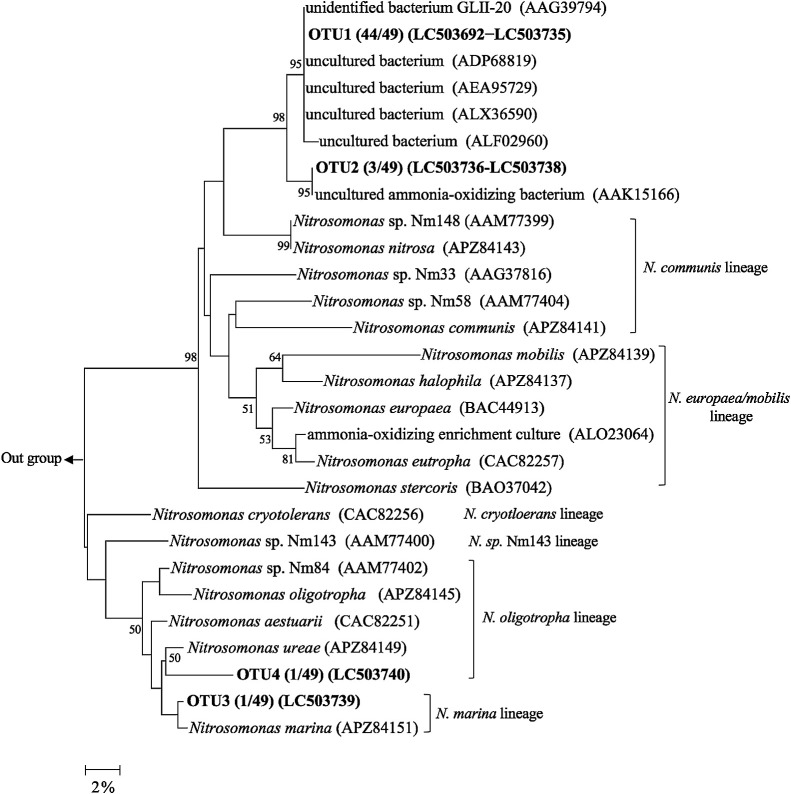
Neighbor-joining phylogenetic tree of betaproteobacterial AmoA amino acid sequences of clones retrieved from BC samples. AmoA of *Nitrosomonas* species and environmental clones detected as the best BLASTx hits were used as reference sequences. *Nitrosospira multiformis* ATCC 25196 was used for the outgroup. The numbers at nodes represent bootstrap values (%, 1,000 resampling); only values greater than 50% are indicated.

Forty-nine clones of the AOB *amoA* gene retrieved from BC samples were randomly selected and sequenced (Table S1), then a NJ phylogenetic tree was constructed using amino acid sequences (Fig. 6). A similar tree was obtained when the nucleotide sequences of the *amoA* gene were used for the phylogenetic calculation (Fig. S2). The tree demonstrated that all of the clones in the present study fell into the genus *Nitrosomonas*, characterized by tolerance to ammonia, which is often detected in the activated sludge of WWTPs (Stehr *et al.*, 1995; Suwa *et al.*, 1997; Koops and Pommerening-Röser, 2001). Higher NH_4_^+^ concentrations in WWTP tanks than in natural environments, such as soils and rivers, may lead to the enrichment of *Nitrosomonas* spp. The sequences of most clones (47/49, OTU1, and OTU2) were not closely affiliated with any lineages of the genus *Nitrosomonas* (*i.e.*, *N. europaea/mobilis* lineage, *N. oligotropha* lineage, *N. communis* lineage, *N. marina* lineage, *N. cryotorelans*, and *N.* sp. Nm143 lineage) (Purkhold *et al.*, 2003; Koops *et al.*, 2006), and formed individual clusters together with uncultured bacterium clones retrieved from freshwater or wastewater samples. The cluster was relatively associated with the *N. europaea/mobilis* lineage. The organisms of this lineage have been found in biofilms in other wastewater treatment systems (Egli *et al.*, 2003; Okabe *et al.*, 2004; Satoh *et al.*, 2006). Moreover, several isolates of this lineage are known to form biofilms with other heterotrophic bacteria (Tsuneda *et al.*, 2001; Petrovich *et al.*, 2017). AOB, which has the ability to form biofilms, may accumulate on BC.

**Fig. 7.**
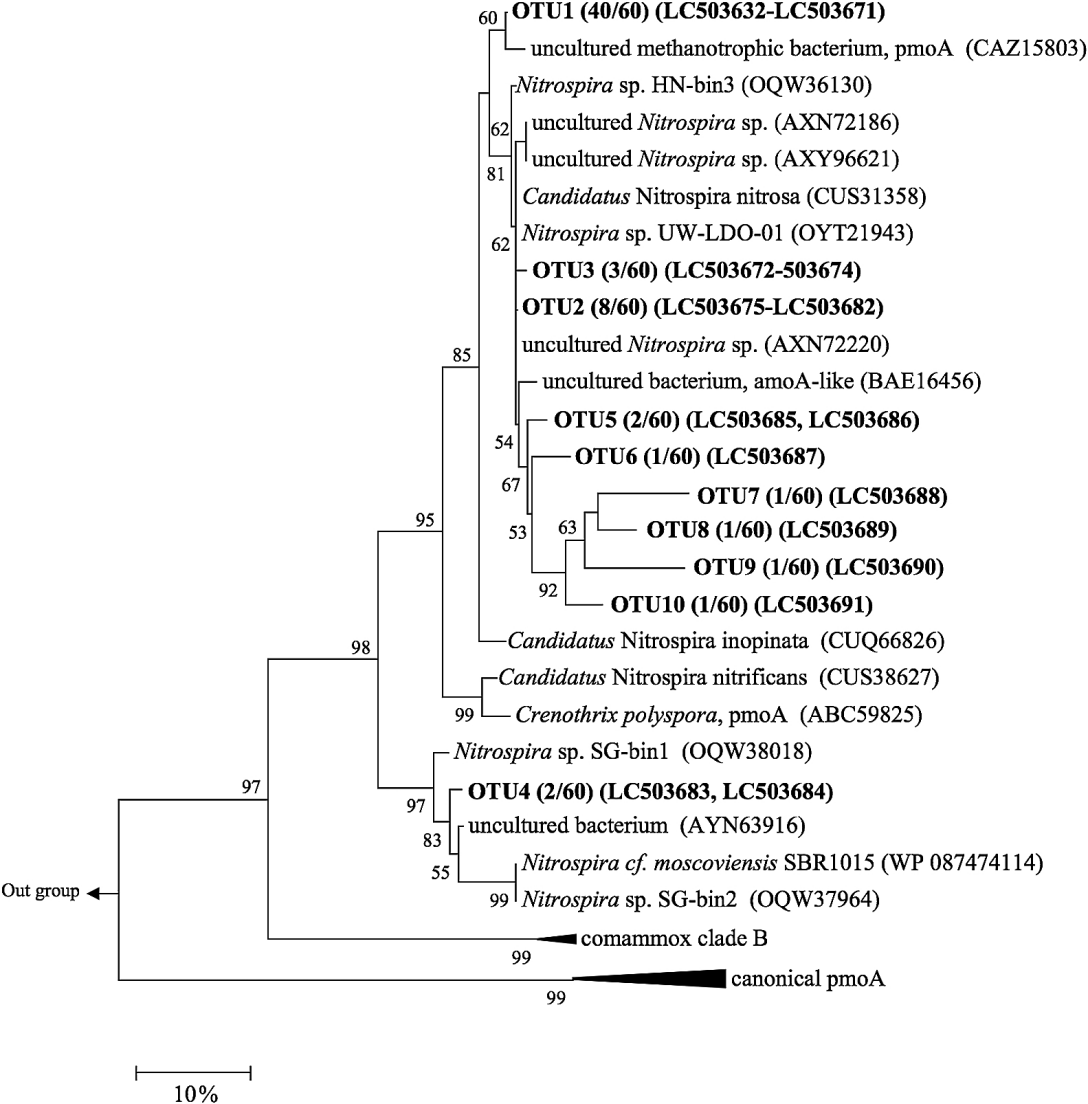
Neighbor-joining phylogenetic tree of comammox AmoA amino acid sequences of clones retrieved from BC samples. AmoA of Comammox *Nitrospira* species and environmental clones detected as the best BLASTx hits were used as reference sequences. *Nitrosospira multiformis* ATCC 25196 was used for the outgroup. The numbers at nodes represent bootstrap values (%, 1,000 resampling); only values greater than 50% are indicated.

PCR cloning of the comammox *amoA* gene retrieved from BC samples was conducted using the primer set for qPCR (Ntsp-amoA 162F and 359R), and the results obtained confirmed that all clone sequences (30 clones) were affiliated with the comammox clade A (data not shown). In order to construct a phylogenic tree using sufficient lengths of the sequences, we used the primer set targeting comammox clade A (COMA-F and -R). Sixty clones of the comammox *amoA* gene (clade A) were sequenced (Table S2), and a NJ phylogenetic tree was constructed using amino acid sequences (Fig. 7). A similar tree was obtained when nucleotide sequences were used for the phylogenetic calculation (Fig S3). The results of the phylogenetic analysis demonstrated that all comammox detected in the present study were classified into the genus *Nitrospira*. The majority of clones (40/60 clones, OTU1) formed individual clusters, which were relatively close to the branch of the AmoA of *Candidatus* Nitrospira inopinata enriched from the biofilms obtained from the walls of deep oil well pipes (Daims *et al.*, 2015) (Fig. 7). This result indicates that as-yet-cultivated comammox *Nitrospira* were predominant in the biofilm. OTU2, OTU3, and OTU5–OTU10 (in total, 18/60 clones) were related to the AmoA of *Candidatus* Nitrospira nitrosa discovered from tap water (van Kessel *et al.*, 2015; Wang *et al.*, 2017) and of uncultured *Nitrospira* sp. clones retrieved from river sediments (Yu *et al.*, 2018). The rest of the clones (2/60 clones, OTU4) were related to *Nitrospira cf. moscoviensis* and the environmental clones of an uncultured bacterium retrieved from river sediments (Xia *et al.*, 2018; Yu *et al.*, 2018). Comammox *Nitrospira* in the biofilm on BC may originate from oligotrophic environments.

As shown in Fig. 7 and Table S2, the particulate methane monooxygenase A (PmoA) of uncultured bacteria were detected as the related sequences of OTUs in the present study. Other studies suggested that the sequences of some of the *pmoA* genes were erroneously classified in databases (van Kessel *et al.*, 2015; Chao *et al.*, 2016; Wang *et al.*, 2018), and should be included in comammox *amoA* based on the findings of phylogenetic analyses (Pinto *et al.*, 2015; Yu *et al.*, 2018). Considering this point, we added the canonical PmoA (alphaproteobacterial, gammaproteobacterial, and verrucomicrobial PmoA) sequences to the tree. As a result, they clustered differently from the comammox AmoA cluster. Therefore, PmoAs detected as relatives of the OTUs of the present study appear to be comammox AmoA.

The rarefaction analysis indicated that the number of species in comammox *Nitrospira* was greater than AOB *Nitrosomonas* in the biofilm (Fig. S4). Various comammox *Nitrospira* species appear to accumulate on BC. To clarify the characteristics of comammox *Nitrospira* on BC, isolation and physiological analyses of comammox *Nitrospira* in natural environments as well as in the biofilm on BC are needed.

## Conclusion

The present study demonstrated the existence of comammox *Nitrospira* in the biofilm formed on carriers suspended in the aerobic tank of a full-scale operational WWTP in Japan. qPCR of *amoA* genes revealed that the abundance of comammox *Nitrospira* in the biofilm was similar to that of AOB* Nitrosomonas*. On the other hand, only AOB *Nitrosomonas* was detected in the activated sludge in the tank, with less abundance than that in the biofilm. Phylogenetic analyses of the *amoA* gene in the biofilm confirmed that as-yet-cultivated comammox *Nitrospira* and uncultured AOB *Nitrosomonas* are predominant in the biofilm. Biofilm formation on carriers appears to be important for the co-existence of comammox and AOB. Further studies are needed to clarify the spatial distribution and activity of ammonia oxidizers inside biofilms on carriers.

## Citation

Tsuchiya, Y., Nakagawa, T., and Takahashi, R. (2020) Quantification and Phylogenetic Analysis of Ammonia Oxidizers on Biofilm Carriers in a Full-Scale Wastewater Treatment Plant. *Microbes Environ ***35**: ME19140.

https://doi.org/10.1264/jsme2.ME19140

## Supplementary Material

Supplementary Material

## Figures and Tables

**Table 1.** PCR primers used in the present study

Target gene	Primer name	Nucleotide sequence (5′-3′)	Position	Reference
AOB *amo*A	amoA-1F	GGGGTTTCTACTGGTGGT	332–349	Rotthauwe *et al.*, 1997
	amoA-2R	CCCCTCKGSAAAGCCTTCTTC	802–822	
COMAMMOX *amo*A (clades A and B, for qPCR)	Ntsp-amoA 162F	GGATTTCTGGNTSGATTGGA	162–182	Fowler *et al.*, 2018
	Ntsp-amoA 359R	WAGTTNGACCACCASTACCA	339–359	
COMAMMOX *amo*A (clade A, for PCR cloning)	COMA F	TGCGGIGACTGGGAYTTC	154–171	Yu *et al.*, 2018
	COMA R	AGATCATAGTGCTRTGICC	649–667	
16S rRNA gene	926F	AAACTCAAAKGAATTGACGG	908–926	De Gregoris *et al.*, 2011
	1062R	CTCACRRCACGAGCTGAC	1981–1064	
