# Supplementary material for: Quantification and Phylogenetic Analysis of Ammonia Oxidizers on Biofilm Carriers in a Full-Scale Wastewater Treatment Plant
Source: Microbes Environ. 2020 Apr 3;35(2):ME19140. doi: 10.1264/jsme2.ME19140 (PMC7308565; doi:10.1264/jsme2.ME19140)
Supplement: Supplementary file 1 — Supplementary Material [file 35_19140_s1.pdf]

Table S1. Phylogenetic information of betaproteobacterial *amoA* OTUs and their corresponding accession number

OTU	Number of clone	Accession number of clone	Closest BLAST match	Accession number of BLAST match
OTU1	44	LC503692-LC503735	Uncultured bacterium clone J70108317_4-51_M13F_13707_C10	MF104002
OTU2	3	LC503736-LC503738	Uncultured ammonia-oxidizing bacterium clone A60441_C28	KP161324
OTU3	1	LC503739	Uncultured bacterium clone GBD A01	KR018127
OTU4	1	LC503740	Uncultured bacterium clone TH0923342_1-13_M13F(-47)	JQ277560

Table S2. Phylogenetic information of comammox *amoA* OTUs and their corresponding accession number

OTU	Number of clone	Accession number of clone	Closest BLAST match	Accession number of BLAST match
OTU1	40	LC503632-LC503671	Uncultured *Nitrospira* sp. clone OTU52	MK290956
OTU2	8	LC503675-LC503682	Uncultured microorganism clone WH16082200144, pmoA	MH638925
OTU3	3	LC503672-LC503674	Uncultured *Nitrospira* sp. clone OTU1	MG387156
OTU4	2	LC503683, LC503684	Uncultured bacterium clone coma_amoA_A-1	MH444516
OTU5	2	LC503685, LC503686	Uncultured *Nitrospira* sp. clone OTU1	MG387156
OTU6	1	LC503687	Uncultured *Nitrospira* sp. clone OTU1	MG387156
OTU7	1	LC503688	Uncultured *Nitrospira* sp. clone OTU1	MG387156
OTU8	1	LC503689	Uncultured *Nitrospira* sp. clone OTU1	MG387156
OTU9	1	LC503690	Uncultured *Nitrospira* sp. clone OTU1	MG387156
OTU10	1	LC503691	Uncultured *Nitrospira* sp. clone OTU1	MG387156

Table S3. The activated sludge and biofilm content in each tank solution

tank	AO	MA	A
activated sludge content (g L^{-1})	39	42	6.9
biofilm content (g L^{-1})	-	-	34

(A)

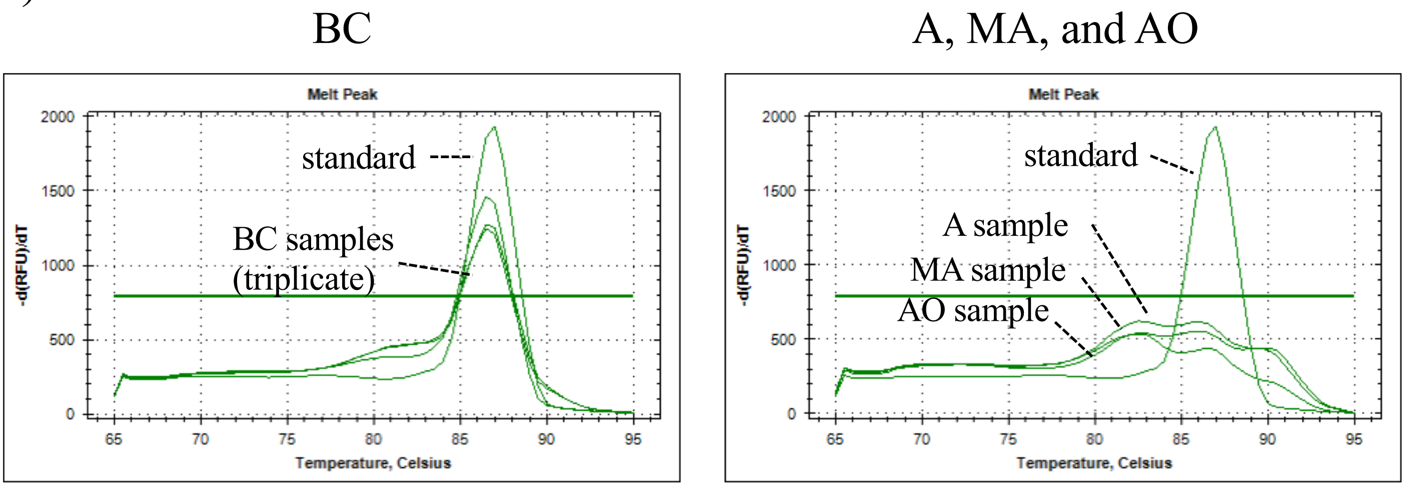

(B)

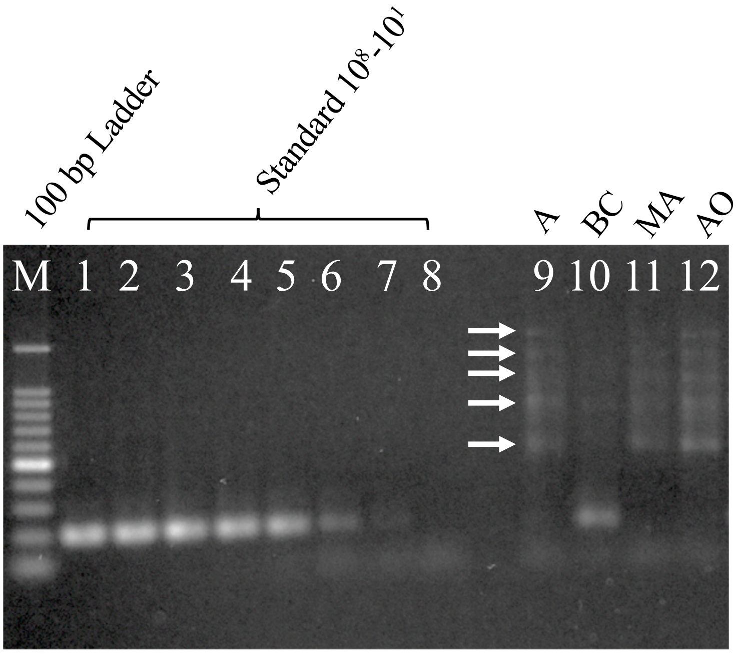

Fig. S1. Melting curve analysis (A) and agarose gel electrophoresis (B) after qPCR of comammox *amoA* gene. Arrows indicate extra bands.

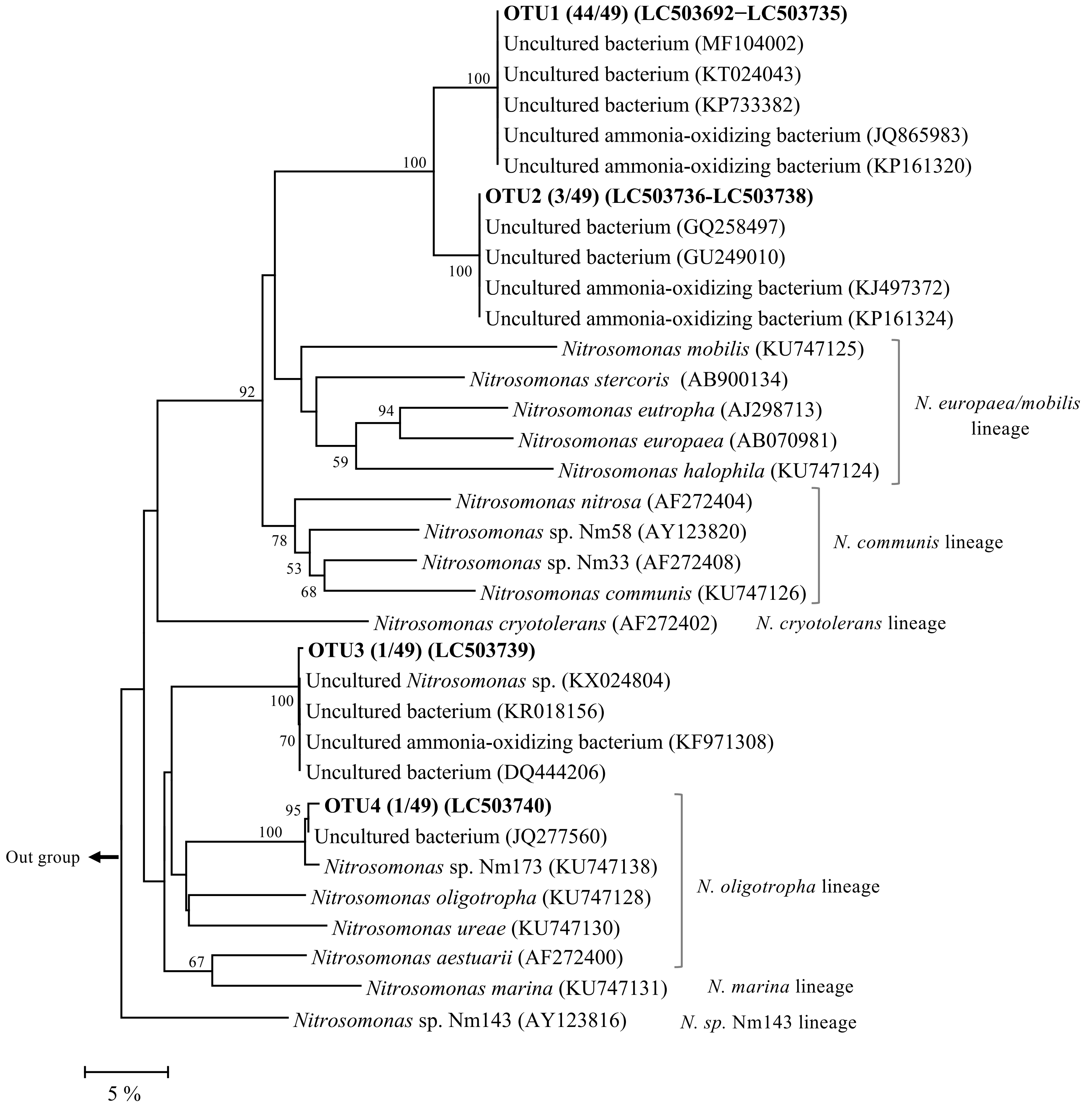

Fig. S2. Neighbor-joining phylogenetic tree of betaproteobacterial *amoA* nucleotide sequences of the clones retrieved from BC samples. *AmoA* of *Nitrosomonas* species and environmental clones detected as the best BLASTn hits were used as reference sequences. *Nitrosospira multiformis* ATCC 25196 was used for the outgroup. The numbers at nodes represent bootstrap values (%, 1000 resampling); only the values greater than 50% are indicated.

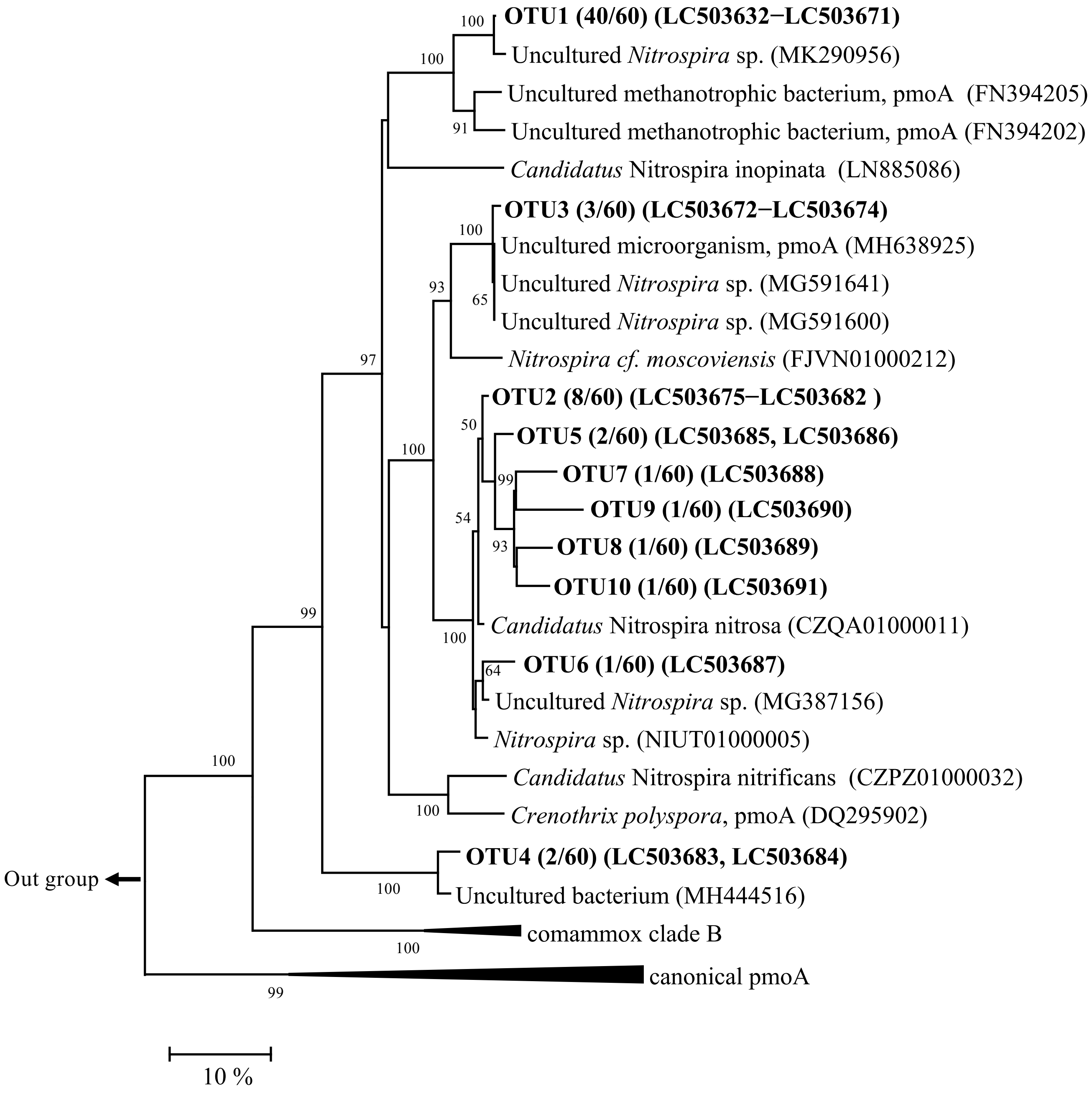

Fig. S3. Neighbor-joining phylogenetic tree of comammox *amoA* nucleotide sequences of the clones retrieved from BC samples. *AmoA* of Comammox *Nitrospira* species and environmental clones detected as the best BLASTn hits were used as reference sequences. *Nitrosospira multiformis* ATCC 25196 was used for the outgroup. The numbers at nodes represent bootstrap values (%, 1000 resampling); only the values greater than 50% are indicated.

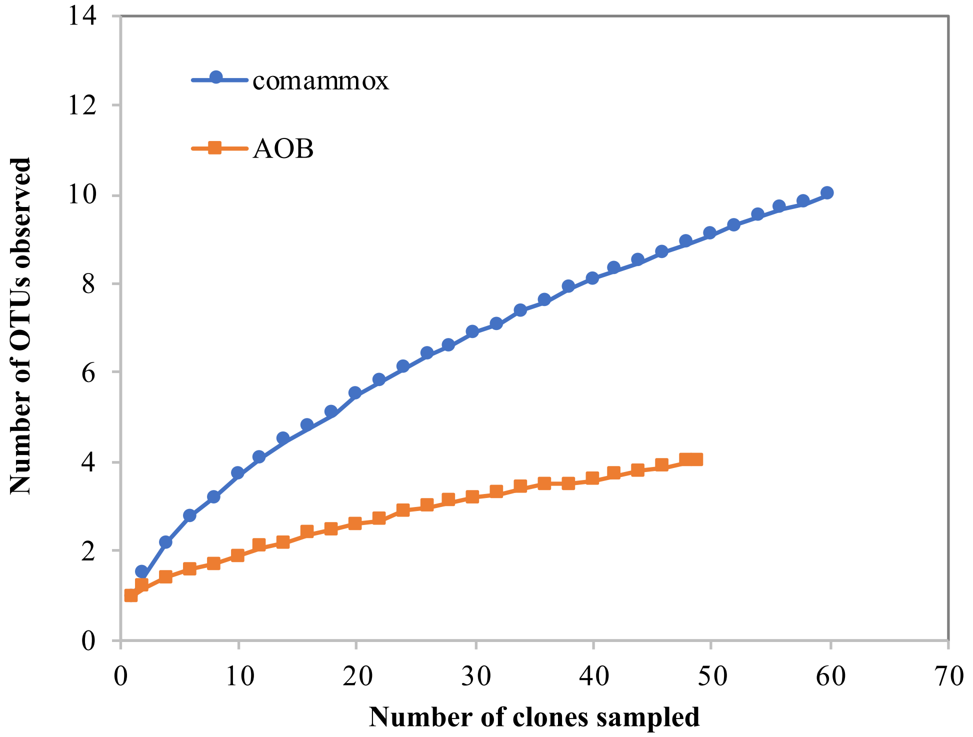

Fig. S4. Rarefaction curve of *amoA* gene clones derived from BC samples. OTU was defined by sequences with 97% similarity.
